# The ISSG-AO Complication Intervention Score, but Not Major/Minor Designation, is Correlated With Length of Stay Following Adult Spinal Deformity Surgery

**DOI:** 10.1177/21925682231202782

**Published:** 2023-09-19

**Authors:** Joseph B. Wick, Andrew Blandino, Justin S. Smith, Breton G. Line, Virginie Lafage, Renaud Lafage, Han Jo Kim, Peter G. Passias, Jeffrey L. Gum, Khaled M. Kebaish, Robert K. Eastlack, Alan Daniels, Gregory Mundis, Richard Hostin, Themistocles Protopsaltis, D. Kojo Hamilton, Michael P. Kelly, Munish Gupta, Robert A. Hart, Frank J. Schwab, Douglas C. Burton, Christopher P. Ames, Lawrence G. Lenke, Christopher I. Shaffrey, Shay Bess, Eric Klineberg, International Spine Study Group

**Affiliations:** 1 Department of Orthopedic Surgery, 8789 University of California, Davis, Sacramento, CA, USA; 2 Department of Neurosurgery, Medical Center, 2358 University of Virginia, Charlottesville, VA, USA; 3 Department of Orthopedic Surgery, Denver International Spine Center, Denver, CO, USA; 4 Department of Orthopedic Surgery, Northwell Health, 5945 Lenox Hill Hospital, New York, NY, USA; 5 Department of Orthopedic Surgery, 25062 Hospital for Special Surgery, New York, NY, USA; 6 Department of Orthopedic Surgery, 12297 NYU Hospital for Joint Diseases, New York, NY, USA; 7 Department of Orthopedic Surgery, Norton Leatherman Spine Center, Louisville, KY, USA; 8 Department of Orthopedic Surgery, 23236 Johns Hopkins University, Baltimore, MD, USA; 9 Department of Orthopedics, San Diego Center for Spinal Disorders, La Jolla, CA, USA; 10 Department of Orthopedics, 6752 Brown University, Providence, RI, USA; 11 Department of Orthopedic Surgery, 192797 Baylor Scoliosis Center, Dallas, TX, USA; 12 Department of Neurosurgery, School of Medicine, 6614 University of Pittsburgh, Pittsburgh, PA, USA; 13 Department of Orthopedic Surgery, 14444 Rady Children’s Hospital, San Diego, CA,; 14 Department of Orthopedic Surgery, 12275 Washington University in St. Louis, St. Louis, MO, USA; 15 Department of Orthopedic Surgery, 7287 Swedish Neuroscience Institute, Seattle, WA, USA; 16 Department of Orthopedic Surgery, 375497 University of Kansas Medical Center, Kansas City, KS, USA; 17 Department of Neurosurgery, 8785 University of California, San Francisco, San Francisco, CA, USA; 18 Department of Orthopedic Surgery, 21611 Columbia University, New York, NY, USA; 19 Department of Neurosurgery, 3065 Duke University Hospital, Durham, NC, USA; 20 Department of Orthopedic Surgery, 12339 University of Texas, Houston, TX, USA

**Keywords:** spine, deformity, complications, length of stay, major, minor, grading, classification, neurologic, adult spinal deformity, scoliosis, degenerative, lumbar, thoracic

## Abstract

**Study Design:**

Retrospective review.

**Objectives:**

The International Spine Study Group-AO (ISSG-AO) Adult Spinal Deformity (ASD) Complication Classification System was developed to improve classification, reporting, and study of complications among patients undergoing ASD surgery. The ISSG-AO system classifies interventions to address complications by level of invasiveness: grade zero (none); grade 1, mild (e.g., medication change); grade 2, moderate (e.g., ICU admission); grade 3, severe (e.g., reoperation related to surgery of interest). To evaluate the efficacy of the ISSG-AO ASD Complication Classification System, we aimed to compare correlations between postoperative length of stay (LOS) and complication severity as classified by the ISSG-AO ASD and traditional major/minor complication classification systems.

**Methods:**

Patients age ≥18 in a multicenter ASD database who sustained in-hospital complications were identified. Complications were classified with the major/minor and ISSG-AO systems and correlated with LOS using an ensemble-based machine learning algorithm (conditional random forest) and a generalized linear mixed model.

**Results:**

490 patients at 19 sites were included. 64.9% of complications were major, and 35.1% were minor. By ISSG-AO classification, 20.4%, 66.1%, 6.7%, and 6.7% were grades 0-3, respectively. ISSG-AO complication grading demonstrated significant correlation with LOS, whereas major/minor complication classification demonstrated inverse correlation with LOS. In conditional random forest analysis, ISSG-AO classification had the greatest relative importance when assessing correlations across multiple variables with LOS.

**Conclusions:**

The ISSG-AO system may help identify specific complications associated with prolonged LOS. Targeted interventions to avoid or reduce these complications may improve ASD surgical quality and resource utilization.

## Introduction

Adult spinal deformity (ASD) is common, with reported prevalence rates as high as 68% among elderly patients.^1^ Symptomatic patients may experience debilitating back pain and neurologic deficits,^2^ resulting in levels of disability similar to other chronic diseases including diabetes, heart disease, and cancer.^3^ Surgical advances have improved surgeons’ ability to correct deformities; however, ASD patients are frequently elderly with multiple comorbidities,^4^ and surgery is associated with complication rates ranging from 37%–71%.^5-9^

Complications are an important metric for assessing and improving surgical quality and safety. Various complication classification systems have been implemented in ASD. Such classification systems include the widely used major/minor complication classification system,^10,11^ as well as the International Spine Study Group (ISSG)-AO ASD Complication Classification System.^12^ In order to be useful, complication classification systems should be tied to clinically meaningful outcomes. Important outcomes include length of stay (LOS), which has been tied to readmission risk,^13^ costs,^14^ and satisfaction^15-18^ among patients undergoing spinal surgery.

While the traditional classification of complications as major/minor is useful for broadly defining complications, such broad definitions may not adequately allow for assessment of complications’ association with important outcome metrics including LOS. In contrast to the major/minor complication classification system, the ISSG-AO system was developed specifically for ASD to allow for more precise complication classification. The granular ISSG-AO complication classification system includes sub-classification of interventions to address complications by level of invasiveness, as follows: Grade zero (none); Grade 1, mild (e.g., medication change); Grade 2, moderate (e.g., ICU admission); Grade 3, severe (reoperation related to surgery of interest).

Given the substantial differences in the major/minor and ISSG-AO ASD complication classification systems, the purpose of this study was to compare whether complications classified by the major/minor and ISSG-AO ASD systems were associated with hospital LOS. We hypothesized that the disease-specific ISSG-AO ASD complication classification system intervention grade sub-component would demonstrate greater correlation with LOS as compared to broadly classifying complications as major/minor.

## Materials and Methods

### Patients and Covariates

Patient data was obtained from a prospective, consecutively enrolled ASD database (ClinicalTrials.gov identifier: NCT00738439). IRB approval was obtained at all 19 US-based sites participating in the ASD database prior to patient enrollment (IRB ID 230538-16). Informed consent was not required due to the retrospective nature of this study. Patients were included in the ASD database if they had a diagnosis of adult degenerative or idiopathic scoliosis with spinal curvature ≥20°, sagittal vertical axis >5 cm, pelvic tilt >25°, thoracic kyphosis >60°, and were age ≥18 at the time of enrollment. Patients were excluded for diagnoses other than degenerative or idiopathic scoliosis or age <18 at the time of surgery or initial consultation. All patients within the database were evaluated for complications sustained during their hospitalization following surgery. Complications identified at post-operative follow-up or necessitating readmission were not included, as these would not affect index LOS. Patients were excluded from the analysis if they were missing data necessary for calculating hospital length of stay or complication intervention grade or severity. The database was reviewed to identify all patients age ≥18 who sustained in-hospital complications following ASD surgery between 2008 and 2020. Multiple datapoints were collected for all patients, including baseline demographics and patient-reported function and pain scores, surgical and perioperative variables, complications including complication treatment intervention, and length of stay (Table 1).

**Table 1.** Baseline, Surgical, and Perioperative Variables.

Variable	n/Median
Baseline demographics	
Total population	490
Age (median years)	64.8
Gender (female)	359 (73.6%)
Prior spine surgery	280 (57.6%)
Pre-operative sensory deficit	25 (5.1%)
Pre-operative chronic pain	47 (9.6%)
BMI	27.4
Baseline depression	133 (27.1%)
Baseline charlson comorbidity index	2
Baseline neurologic motor/sensory deficits	294 (62.0%)
ASA grade	
1	20 (4.2%)
2	208 (43.2%)
3	245 (50.9%)
4	8 (1.7%)
Baseline frailty index score (median)	3.6
Institution/site	19 total
Baseline patient reported function scores (medians)	
Oswestry disability index score	48
SF-36 PCS score	28.2
SF-36 MCS score	46.1
SF-36 physical functioning	25.5
SF-36 role physical	27.5
SF-36 bodily pain	29.2
SF-36 general health	45.8
SF-36 vitality score	39.6
SF-36 social function	35
SF-36 role emotional	40.3
SF-36 mental health	44.3
SRS total score	2.7
SRS activity score	2.6
SRS pain score	2.2
SRS appearance score	2.4
SRS mental health score	3.4
SRS satisfaction score	2.5
Surgical/perioperative variables	
Surgical approach	
Anterior only	2 (.4%)
Posterior only	316 (64.8%)
Anterior-posterior	169 (34.6%)
MIS only	1 (.2%)
ISSG level of surgical invasiveness (median)	96.5
Operative time (minutes, median)	391
Estimated blood loss (median mL, first stage for staged procedure)	1500
Estimated blood loss (mL, total, median)	1800
First stage anterior approach	73 (14.9%)
First stage posterior approach	403 (82.4%)
First stage interbody fusion	326 (66.7%)
First stage decompression	255 (52.1%)
First stage osteotomy	312 (63.8%)
First stage post-operative ICU admission	335 (69.2%)
Any decompression performed	300 (61.3%)
Any osteotomy performed	370 (75.8%)
Complications/outcomes	
Length of stay (median days)	8
First stage SSEP neuromonitoring change	29 (6.2%)
First stage MEP change	31 (6.7%)
ISSG complication intervention classification	
Grade 0	100 (20.4%)
Grade 1	324 (66.1%)
Grade 2	33 (6.7%)
Grade 3	33 (6.7%)
Major/minor complication classification (for most severe complication)	
Major	346 (70.6%)
Minor	144 (29.4%)
Multiple complications	176 (35.9%)

Abbreviations: BMI, Body Mass Index; ASA, American Society of Anesthesiologists; SF-36, Short-Form 36 item Survey; SRS, Scoliosis Research Society; PCS, physical component summary; MCS, mental component summary; MIS, minimally invasive surgery; ISSG, International Spine Study Group; ICU, intensive care unit; SSEP, somatosensory evoked potentials; MEP, motor evoked potentials.

### Complication Grading

**Figure 1.**
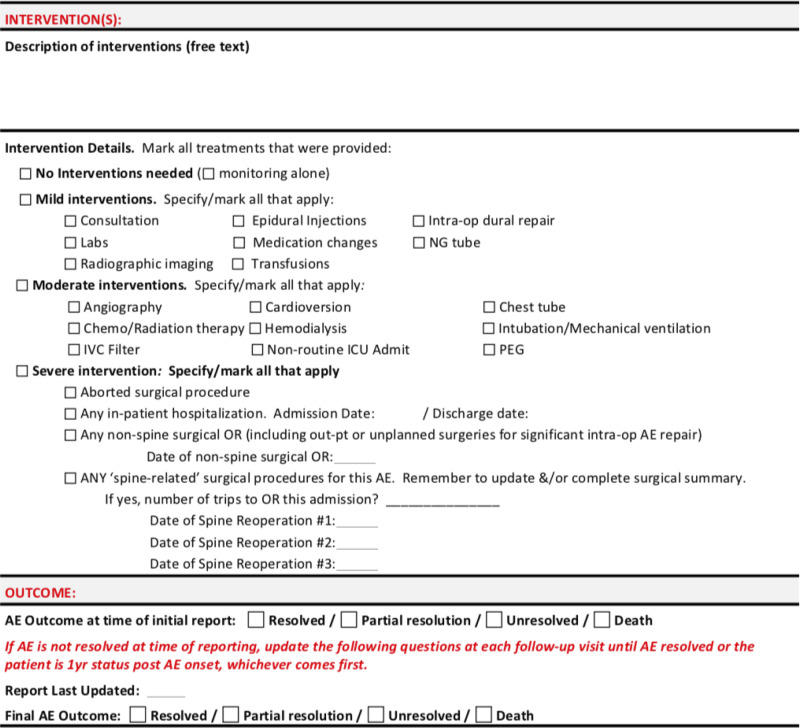
ISSG-AO complication classification system grading worksheet. The “Intervention Details” section was used to grade the intervention severity for each complication, as follows: No interventions = Grade 0, Mild interventions = Grade 1, Moderate interventions = Grade 2, Severe interventions = Grade 3.

All complications were graded as major/minor^10,11^ (Table 2) and by the level of complication intervention invasiveness/severity based on the ISSG-AO ASD complication classification system definitions (Figure 1). This study specifically evaluated the ISSG-AO classification system’s complication intervention severity component, which assigns complication intervention severity grade based solely on the intervention utilized to address the complication (Figure 1).^12^ For example, a superficial wound infection requiring antibiotic treatment would be graded as 1 (mild intervention requiring medication change) whereas deep wound infection requiring irrigation and debridement would be graded as 3 (severe intervention requiring return to OR). Similarly, complications necessitating additional intraoperative or operative procedures, such as vascular injury requiring repair, are graded as 3. Neurologic changes treated with medications are graded as 1, whereas neurologic changes treated with additional surgical decompression are graded as 3. For patients sustaining multiple complications, all complications were graded as separate events. Complication outcomes, including deaths, are accounted for separately from the complication intervention severity, and do not directly influence the complication intervention severity grade. Complication intervention severity grading was initially performed by the submitting surgeon, and was then reviewed by the ISSG complication committee, comprised of three fellowship-trained spine surgeons. Complication grading was then confirmed by the senior author, who is a fellowship-trained orthopedic spine surgeon (EK).

**Table 2.** Major/Minor Classification of Surgical Complications.

Category	Complication description	
	Intra-operative	Post-operative before discharge
Major	Bowel/bladder deficit	Bowel/bladder deficit
	Cardiac arrest	Death
	Cauda equina deficit	Deep vein thrombosis
	Cord deficit	Infection- deep
	Death	Motor deficit
	Inadvertent extubation	Myocardial infarction
	Malignant hyperthermia	Neurological complications
	Nerve root injury	Optic deficit
	Optic deficit	Pneumonia
	Vascular injury	Pulmonary embolism
	Visceral injury	Reintubation
		Sepsis
		Stroke
		Other cardiopulmonary complication
Minor	Cerebrospinal fluid leak	Infection- superficial
	Excessive bleeding	Postoperative radiculopathy
	Ineffective fixation	Sensory deficit
	Intraoperative coagulopathy	Skin complications
	Pedicle infraction	Excessive postoperative bleeding
	Posterior element fracture	Thrombophlebitis - superficial
	Vertebral body fracture

### Univariate Comparisons

Initial univariate analyses were performed to assess correlation of variables with LOS and to assess the effect of patients sustaining multiple complications. To assess the effect of multiple complications of varying severity on LOS, Kruskal-Wallis tests were used to compare LOS vs complication grade and LOS vs major/minor complications. Specifically, comparisons were performed between patients with and without multiple complications by evaluating patients based on their least severe, median severity, and most severe complications. Among those sustaining multiple complications, “median grade” and “median severity” were calculated for the ISSG-AO ASD complication classification system and the major/minor complication systems, respectively. For the ISSG-AO complication classification system, median complication grade was based on the median intervention grade of all complications sustained by the patient. Similarly, for the major/minor system, median complication severity was determined by whether patients sustained a greater number of minor or major complications.

### Multivariate Comparisons

Two multivariate modeling approaches were employed to further assess associations with LOS. In the first model, a conditional random forest machine learning algorithm analyzed relative importance of each demographic, surgical, and perioperative variable in association with LOS. To account for patients sustaining multiple complications, the model was repeated to assess relative importance of ISSG-AO complication grade and major/minor classification in relation to patients’ least severe, median severity, and most severe complication. The conditional random forest model also included a “multiple complications” variable to account for patients who sustained more than one complication.

A generalized linear mixed model using a Poisson distribution with a log link was constructed. The hospital/participating center for each surgery was considered as a random effect to account for clustering of patients within the same hospital. Variables from the univariable analysis were included as potential confounders if their association with LOS was significant at *P* < 0.1. Separate generalized linear mixed models were constructed to assess fit for LOS against ISSG-AO grade and adjustment variables, as well as fit for LOS against major/minor and adjustment variables. For complication severity grade using the ISSG-AO system, additional pairwise comparisons were performed by grouping complication grades and comparing LOS between these groups.

All analyses were performed by an institutional statistician using R, version 4.1.2 (https://www.r-project.org). Level of significance was set at *P* = .05.

## Results

### Patients and Covariates

The ISSG database includes a total of 1999 participants. A total of 616 patients were identified in the database as having sustained one or more in-hospital complications and were reviewed for study inclusion (Table 1). Patients were excluded if they were missing complication status information, data necessary for complication grading, or if patients had incomplete hospital admission and discharge data precluding LOS calculation. Of the 616 patients, a total of 490 had complete data and were included in the final analysis. Among included patients, 73.6% were female, median age was 64.8 years, and 57.6% of patients had a history of prior spine surgery. Median LOS was 8 days. When classified by the ISSG-AO system, 20.4% of complications were Grade 0, 66.1% were Grade 1, 6.7% were Grade 2, and 6.7% were Grade 3. When classified by the major/minor complication classification system, 70.6% were major and 29.4% were minor. A total of 35.9% of patients sustained multiple complications.

### Univariate Comparisons

**Figure 2.**
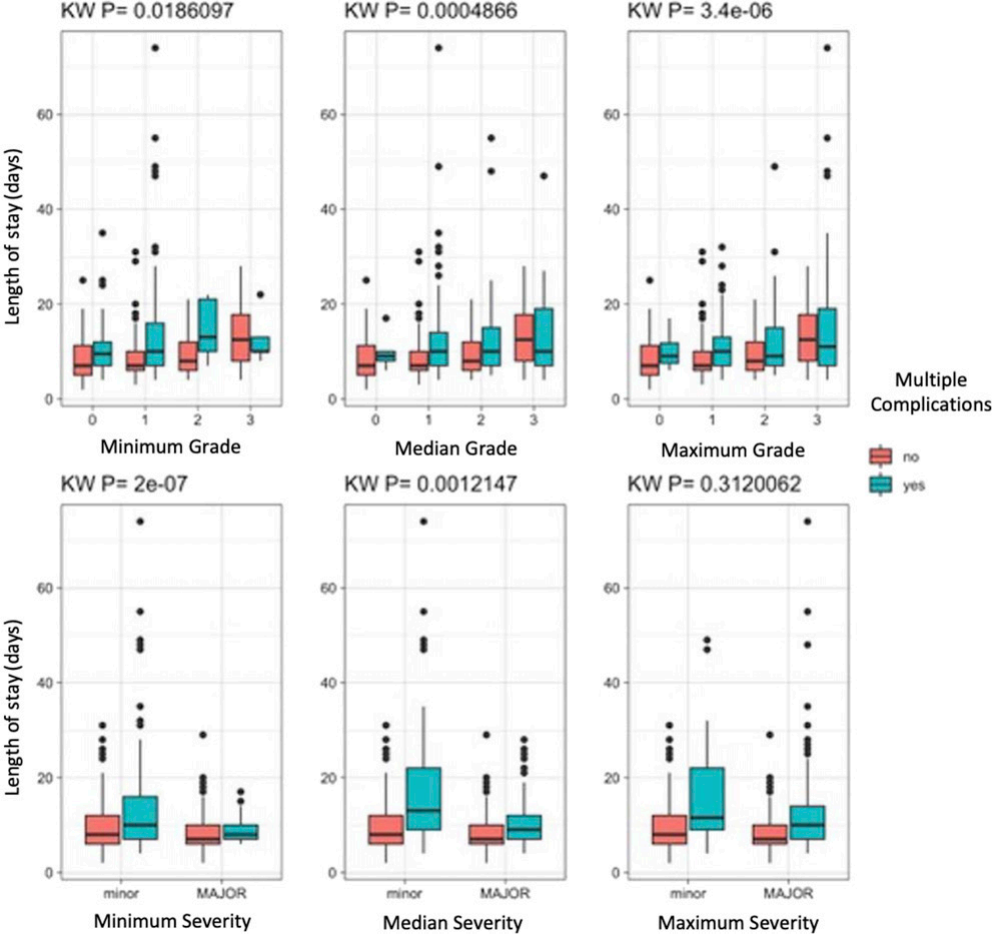
Kruskal-Wallis comparisons between patients with and without multiple complications, with comparisons performed for complications graded using the ISSG-AO ASD complication classification system (top row) and major/minor classification system (bottom row). For those sustaining multiple complications, “median grade” represents the median value of complication intervention severity of all complications sustained by patients, as determined based on the ISSG-AO complications classification system. “Median severity” represents the median value of all major/minor complications sustained by patients, as determined based on major/minor complications classification. KW value represents *P*-value, with level of significance at *P* = .05.

Figure 2 shows results of Kruskal-Wallis comparisons between patients with and without multiple complications. Increasing complication severity as classified by the ISSG-AO system correlated with increased LOS regardless of whether patients sustained one or multiple complications. The strongest correlation was identified between LOS and most severe complication as classified by the ISSG-AO system, with *P* = 3.4 × 10^−6^.

When classified as major/minor, longer LOS was correlated with minor rather than major complications when patients were assessed based on their least severe and median severity complication (p = 2 × 10^−7^ and *P* = 1.2 × 10^−3^, respectively). No correlation was identified between major/minor complication severity and LOS when patients were evaluated based on their most severe complication sustained.

### Multivariate Modeling and Pairwise Comparisons

**Figure 3.**
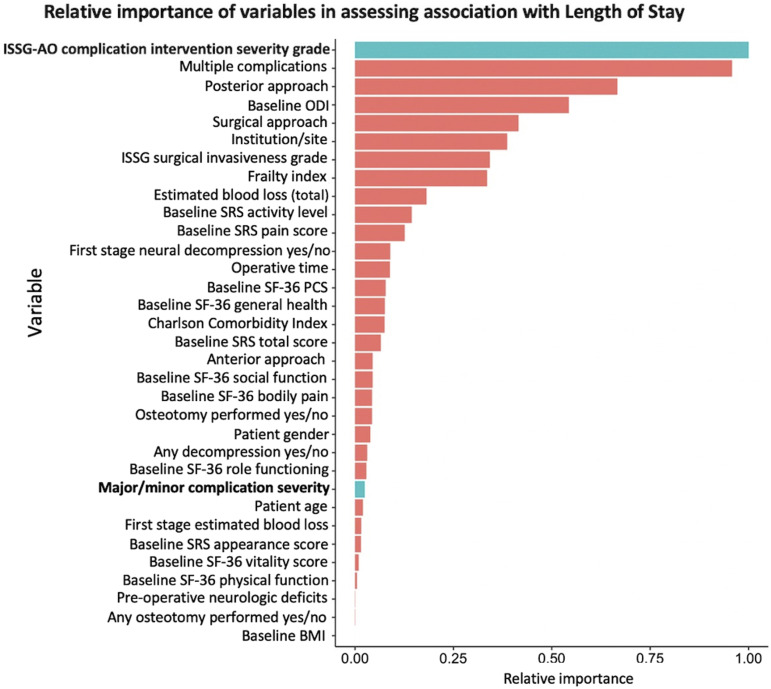
Conditional random forest machine learning algorithm analyzing relative importance of association between variables and LOS when patients were evaluated based on their most severe complication. Abbreviations: ODI, Oswestry Disability Index; ISSG, International Spine Study Group; SRS, Scoliosis Research Society; SF-36, Short Form 36-Item Survey; BMI, Body Mass Index.

Figure 3 shows results of the conditional random forest machine learning algorithm analyzing relative importance of association between variables and LOS when patients were evaluated based on their most severe complication. For this analysis, ISSG-AO grade was found to have the highest relative importance, approaching 1.0 in relation to LOS, followed in decreasing order by multiple complications, posterior approach, and baseline disability. Classification of complications as major/minor had much less importance in relation to LOS, with a relative value of <0.1.

**Figure 4.**
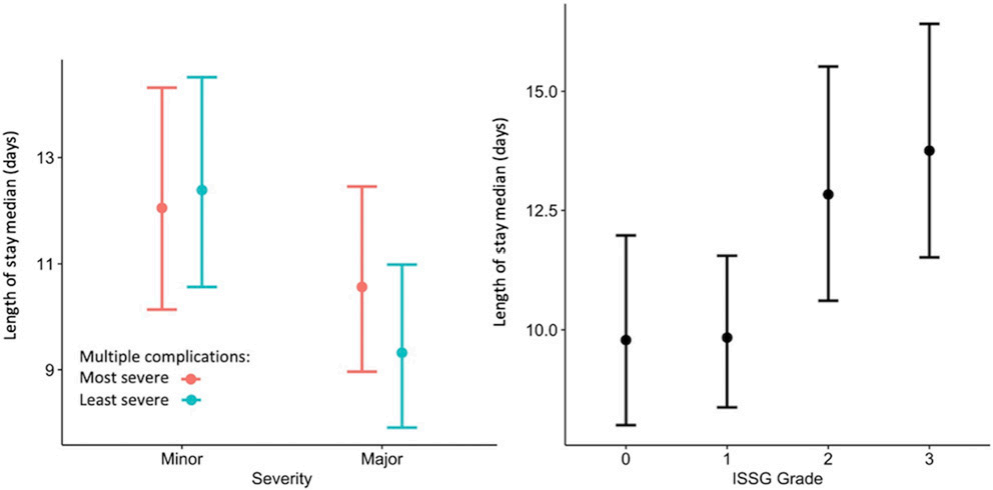
Generalized linear mixed models fit for length of stay vs complication severity graded by major/minor classification (left panel) and ISSG grade (right panel). Fit for major/minor classification was repeated based on patients’ most severe and least severe complications. Abbreviations: ISSG, International Spine Study Group.

Figure 4 shows results of generalized linear mixed models. When fit for LOS vs ISSG-AO grade and adjustment variables (Table 3), the model demonstrated significant correlation between increased ISSG-AO complication grade and LOS, with *P* = .0027 for Grade 2, and *P* < .001 for Grade 3 complications. Female gender, level of invasiveness, and baseline general health were additionally found to correlate significantly with LOS. In contrast to ISSG-AO grade, general linear mixed model fit for LOS vs major/minor complication classification and adjustment variables (Table 4) found a significant, inverse correlation between increased complication severity (major complication as opposed to minor complication), with coefficient of -.1313 and *P* = .012. In this analysis, performance of an osteotomy during the first stage of surgery, level of surgical invasiveness, and baseline SF-36 general health were also found to correlate with increased LOS.

**Table 3.** Generalized Linear Mixed Model Assessing Fit for Length of Stay Versus Categorical ISSG-AO Grade and Adjustment variables.

Variable	Coefficient	Standard error	*P*-value
(Intercept)	2.5508	.1752	0
ISSG-AO grade 1	.0052	.0763	.946
ISSG-AO grade 2	.2712	.0905	**.003**
ISSG-AO grade 3	.3404	.0891	**<.001**
First stage posterior approach	-.159	.0942	.092
First stage osteotomy	-.1624	.0945	.086
First stage decompression	-.0493	.0539	.361
First stage interbody fusion	-.0075	.0530	.888
ASA grade 2	-.0674	.1207	.576
ASA grade 3	-.1416	.1257	.260
ASA grade 4	-.1153	.2040	.572
Any osteotomy performed	.113	.0919	.219
Female gender	-.1126	.0510	**.027**
ISSG level of surgical invasiveness	.0977	.0245	**<.001**
SF-36 general health	-.0742	.0292	**.011**
Estimated blood loss (total)	.0423	.0243	.082
Baseline frailty index (median)	.0072	.0441	.871
Baseline oswestry disability index score	.0158	.0363	.664
Baseline SRS appearance score	.0019	.0283	.947
Baseline age	.0493	.0254	.052

Abbreviations: ISSG-AO, International Spine Study Group-AO foundation; ASA, American Society of Anesthesiologists; SF-36, Short-Form 36 Item Survey; SRS, Scoliosis Research Society. Significant associations are in bold, with P < 0.05.

**Table 4.** Generalized Linear Mixed Model Assessing Fit for Length of Stay Versus Major/Minor Classification for Most Severe Complication Sustained and Adjustment variables.

Variable	Coefficient	Standard error	*P*-value
(Intercept)	2.7815	.1754	—
Major complication	–.1313	.0511	**.010**
First stage posterior approach	–.1823	.0977	.062
First stage osteotomy	–.2008	.0977	**.040**
First stage decompression	–.038	.0557	.496
First stage interbody fusion	–.0041	.0546	.941
ASA grade 2	–.1046	.1245	.401
ASA grade 3	–.1552	.1297	.232
ASA grade 4	–.1118	.2107	.596
Any osteotomy performed	.1578	.0953	.098
Female gender	–.1322	.0531	.013
ISSG level of surgical invasiveness	.1055	.0254	**<.001**
SF-36 general health	–.0674	.0303	**.026**
Estimated blood loss (total)	.0284	.0252	.261
Baseline frailty index (median)	–.0163	.0456	.722
Baseline oswestry disability index score	.0308	.0374	.411
Baseline SRS appearance score	–.0092	.0291	.752
Baseline age	.0467	.0261	.074

Abbreviations: ISSG-AO, International Spine Study Group-AO foundation; ASA, American Society of Anesthesiologists; SF-36, Short-Form 36 Item Survey; SRS, Scoliosis Research Society. Significant associations are in bold, with *P* < 0.05.

Additional pairwise comparisons evaluating ISSG-AO complication grades and LOS are shown in Table 5. Significant differences were found between complication grades zero and 1 vs grades 2 and 3.

**Table 5.** Pairwise Comparisons Between ISSG-AO Complication Grades and Association With Length of Stay.

ISSG-AO Grades comparison	Ratio	Standard Error	*P*-value
Grade zero vs grade 1	.995	.076	.999
Grade zero vs grade 2	.762	.070	**.014**
Grade zero vs grade 3	.711	.063	**<.001**
Grade 1 vs grade 2	.766	.050	**<.001**
Grade 1 vs grade 3	.715	.044	**<.001**
Grade 2 vs grade 3	.933	.074	.821

Abbreviations: ISSG-AO, International Spine Study Group-AO Foundation. Significant associations are in bold, with P < 0.05.

## Discussion

The ISSG-AO ASD complications classification system was recently introduced to improve classification and understanding of postoperative complications specific to ASD patients. To assess the efficacy of the ISSG-AO classification system, we aimed to identify whether ISSG-AO complication intervention scoring correlates better with post-operative LOS as compared to the traditionally used major/minor complication classification system. Understanding associations with LOS is especially important, as increased LOS has been associated with higher readmission risk,^13^ increased costs,^14^ and diminished satisfaction^15-18^ among patients undergoing spinal surgery. We evaluated a large, multicenter cohort of 490 ASD patients sustaining in-hospital complications and classified the complications with both the ISSG-AO and major/minor systems. Multiple statistical approaches confirmed significant correlation between ISSG-AO complication severity classification and LOS, whereas the major/minor classification system showed an inverse relationship between complication severity and LOS.

Results of our study suggest that the ISSG-AO ASD complication intervention grading is a reliable means for identifying complications associated with prolonged LOS. This association was found to be independent of other factors, including staged surgery, patient frailty, and other comorbidities. Specifically, we found that the complication intervention sub-classification of the otherwise granular ISSG-AO classification system score strongly correlates with LOS, with grades 2 and 3 correlating most strongly with prolonged LOS in pairwise comparisons. Use of the complication intervention sub-classification may simplify evaluation of complications influencing LOS, especially as the ISSG-AO system’s 0-3 grading scale for complication intervention severity (Grade 0, none; Grade 1, mild; Grade 2, moderate; Grade 3, severe) is straightforward, with clear guidelines for each complication grade (Figure 1).^12^ In addition, previous work has shown the ISSG-AO complication classification system to be highly repeatable, with good inter- and intra-rater reliability across varying levels of training, including those with and without surgical training. Indeed, overall intervention severity grading, or ability to correctly choose complication intervention severity grade, was 88.4% correct among all raters, including fellowship trained surgeons, residents/fellows, and research coordinators.^12^ Furthermore, our results show that the widely used major/minor classification system cannot reliable correlate complication types with LOS, further supporting the need for an ASD-specific complications classification system. As such, the major/minor classification scheme appears to inadequately account for severity of complications and their implications as they relate to clinically meaningful outcomes such as LOS. For example, minor complications include cerebrospinal fluid leak and inadequate fixation, which may require additional procedures such as re-exploration or revision fixation. Such additional procedures may be expected to substantially prolong length of stay, and therefore may not be truly “minor” complications as they have meaningful implications for patients, payors, and other health care stakeholders.

To our knowledge, this is the first study to compare the correlation of the ISSG-AO ASD complications classification system grading to major/minor complication grading. We specifically chose to compare the ISSG-AO system to the major/minor system, as the major/minor system has been widely used for evaluating complications in ASD surgery. While our results show correlation between complication intervention severity and LOS, our study was not intended to provide a predictive model for LOS. Similarly, while previous studies have shown correlations between factors such as frailty and complications,^19^ we were not specifically evaluating predictors of complications. Our results build on prior work by Le et al, who reported that increased ISSG-AO complication severity was significantly correlated with longer LOS among ASD patients. Similar to our study, Le et al found significant association between ISSG-AO complication grade and LOS after adjusting for multiple factors, including staged surgery and frailty.^20^ In contrast, prior studies have not consistently correlated LOS and increased complication grade using alternative classification systems, such as the Clavien-Dindo (CD) grading system.^21^ The CD system is similar to the ISSG-AO system in grading complications based on complication treatment intervention; however, the CD system was developed for in-hospital complications in general surgery rather than complications specific to ASD.^20,22-24^ Studies have also found no correlation between CD complication grading and LOS in patients undergoing surgery for cervical deformity,^24^ further emphasizing the need for spine- and ASD-specific complication classification systems.

Disease-specific complication classification systems such as the ISSG-AO ASD system will likely play an increasingly important role in outcomes research with the aim of improving health care delivery. While the aim of the current study was validating the ISSG-AO ASD complications classification system and comparing the ISSG-AO system to the widely used major/minor system, it is important to note that other spine-specific complication classification systems have been validated, including the Spine Adverse Events Severity System (SAVES).^25,26^ Development and application of such systems is especially important as health care, and spine surgery in particular, faces increased pressure to improve costs, quality, and outcomes.^27^ With our study, we have shown that ISSG-AO complication grading correlates strongly with LOS. Widespread implementation of accurate, disease specific complication classification systems such as the ISSG-AO system may facilitate communication between health care stakeholders and ultimately improve our understanding of complication risk factors and their consequences, including prolonged LOS. In turn, improved understanding of complications may help surgeons identify patients at risk for complications and diminished outcomes, improve preoperative patient selection and counseling, set appropriate expectations and surgical goals, including extent of surgery, and mitigate risks for poor outcomes by pursuing appropriate preoperative patient optimization.^28^

This study was limited by its retrospective design, lack of a control group, and lack of a standardized discharge protocol among included centers. We also do not have data on the proportion of patients reported to the database from each center. We evaluated patients who underwent surgery between 2008 and 2020, and our data may be subject to changes in practices and protocols over time. In turn, this may limit generalizability of our results. We were also unable to include long-term outcomes or cost data. We did not account for time between procedures for staged surgery; however, we assessed and accounted for surgical approach including combined anterior-posterior approach, which may serve as a surrogate for staged surgery. Furthermore, while Klineberg et al found a high degree of complication intervention severity grading accuracy with the ISSG-AO system,^12^ we were unable to assess the accuracy or reliability of data submitted from participating centers, or the accuracy of the complication intervention grading within our dataset. Despite these limitations, this is the first study to specifically compare the traditionally used major/minor complications classification system to the ASD-specific ISSG-AO complications classification system. We analyzed data from many patients and surgeons across 19 sites**.** While we did not assess for surgeon-level variability, our analysis accounted for clustering of patients among centers to minimize the effect of local practice patterns and improve overall generalizability.

## Conclusion

Our findings demonstrate that the ISSG-AO ASD complications classification intervention severity grading correlates strongly with increased LOS, whereas the traditionally used major/minor classification system had an inverse correlation with complication severity and LOS. Widespread implementation of the ISSG-AO ASD complication classification system may help spine surgeons better understand complications, their risk factors, and clinical implications, ultimately helping surgeons mitigate complications and improve the overall value of ASD care.
